# Compound Heterozygous Variants in Pediatric Cancers: A Systematic Review

**DOI:** 10.3389/fgene.2020.00493

**Published:** 2020-05-19

**Authors:** Dustin B. Miller, Stephen R. Piccolo

**Affiliations:** Department of Biology, Brigham Young University, Provo, UT, United States

**Keywords:** pediatric cancer, germline variants, compound heterozygosity, variant pathogenicity assessment, genetic analysis of complex diseases

## Abstract

A compound heterozygous (*CH*) variant is a type of germline variant that occurs when each parent donates one alternate allele and these alleles are located at different loci within the same gene. Pathogenic germline variants have been identified for some pediatric cancer types but in most studies, *CH* variants are overlooked. Thus, the prevalence of pathogenic *CH* variants in most pediatric cancer types is unknown. We identified 26 studies (published between 1999 and 2019) that identified a *CH* variant in at least one pediatric cancer patient. These studies encompass 21 cancer types and have collectively identified 25 different genes in which a *CH* variant occurred. However, the sequencing methods used and the number of patients and genes evaluated in each study were highly variable across the studies. In addition, methods for assessing pathogenicity of *CH* variants varied widely and were often not reported. In this review, we discuss technologies and methods for identifying *CH* variants, provide an overview of studies that have identified *CH* variants in pediatric cancer patients, provide insights into future directions in the field, and give a summary of publicly available pediatric cancer sequencing data. Although considerable insights have been gained over the last 20 years, much has yet to be learned about the involvement of *CH* variants in pediatric cancers. In future studies, larger sample sizes, more pediatric cancer types, and better pathogenicity assessment and filtering methods will be needed to move this field forward.

## Introduction

Each year worldwide, ~300,000 children under the age of 14 are diagnosed with cancer (Sweet-Cordero and Biegel, 2019). Since the 1970s, 5-year survival rates for pediatric cancer patients have steadily increased and are presently over 80% (Phillips et al., 2015). Despite improvements in treatments and survival rates, the causes of most pediatric cancers are still relatively unknown (American Cancer Society^1^). Recent large-scale studies have helped elucidate the involvement of germline mutations in pediatric cancer development. For example, a 2015 study analyzed mutations across 565 known, cancer-associated genes for 1,120 pediatric cancer patients and found that 8.5% of the patients had an identifiable, pathogenic germline mutation in at least one of these genes (Zhang et al., 2015). Similarly, a 2016 study identified 10% of pediatric cancer patients as having a mutation in a cancer predisposition gene (Parsons et al., 2016). These studies highlight that heritable predisposition does play a critical role in many pediatric cancer cases (Dean and Farmer, 2017). However, these studies also emphasize the need to elucidate additional types of rare, germline variants associated with pediatric cancers. In this review, we focus on *compound heterozygous* (*CH*) variants, a type of germline variant that has been understudied in pediatric cancers.

**Figure 1:**
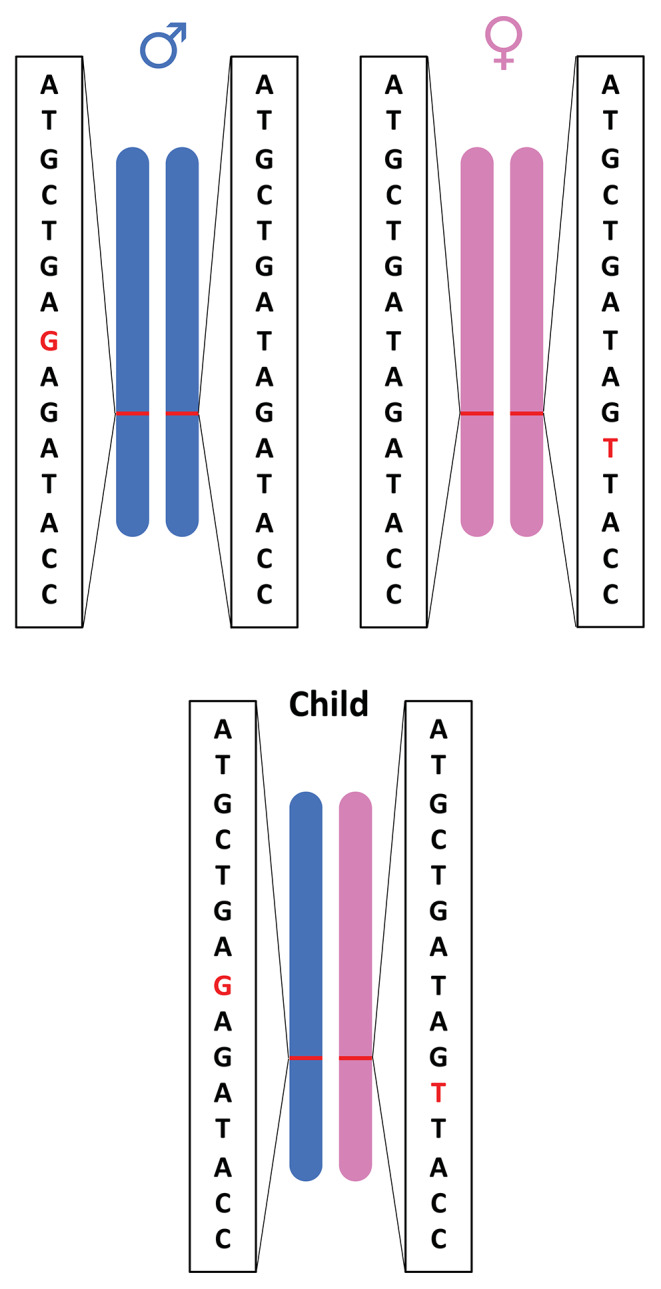
Illustration of compound heterozygous variants. Compound heterozygous variants occur when a child has an alternate allele from each parent and the variant is located at different loci within the same gene.

*CH* variants occur when each parent donates one alternate allele and when the alleles are located at different loci within the same gene (Figure 1; Kamphans et al., 2013). *CH* variants are particularly relevant to certain types of genes, such as tumor suppressors, where loss-of-function variations are often recessive (Wang et al., 2018). Tumor suppressor genes are involved in inhibiting cell division, initiating apoptosis, repairing DNA damage, and suppressing metastasis. When tumor suppressor genes lose function, tumors can arise and existing tumors can become more aggressive (Guo et al., 2014; Wang et al., 2018). Commonly, researchers identify cases where two non-reference alleles at a given genomic locus have been inherited, one from each parent (homozygosity). But in other cases, an individual may inherit two defective copies of a tumor suppressor gene—one from each parent, with defects at different loci in the gene—thereby resulting in no functional copies of the tumor suppressor gene and potentially an increased susceptibility to cancer development (Weinberg, 2013).

Sanger sequencing has classically been used to identify *CH* variants. In this approach, DNA is collected from the patient and his/her parents, and visual analysis of an electropherogram helps to confirm Mendelian inheritance of a *CH* variant (Piane et al., 2016; Nafisinia et al., 2017). These technologies are most relevant when the researcher wishes to examine one or a few genes. In recent years, detection of *CH* variants at a larger scale has become feasible with the advent of next-generation sequencing (NGS).

NGS technologies (e.g., Illumina, PacBio) can sequence DNA in a high-throughput manner and thus allow for the examination of many genes in a single run (Tewhey et al., 2011). In order to use NGS data for identifying *CH* variants, sequencing data must be phased. The process of phasing estimates which chromosome (haplotype) the nucleotides are located on, thereby helping to distinguish between maternally and paternally inherited variants (Choi et al., 2018). If it is known before sequencing that haplotype information will be needed, a laboratory-based, haplotype-estimation method can be used, such as Linked-Read technology by 10X Genomics (Zheng et al., 2016). This approach uses micro-droplet-based dilution to compartmentalize DNA in a random manner and uses a high number of distinct barcodes. This method prevents the partitioned DNA molecules from originating from the same genomic loci. Alternatively, if NGS libraries have been prepared without regard to phase, computer-based phasing algorithms can be used to estimate haplotypes (Browning and Browning, 2007; Delaneau et al., 2013; Loh et al., 2016). These algorithms estimate a patient's haplotypes from genotype data after making inferences from a population-based reference panel and/or inferences from parental inheritance patterns.

Assessment of *CH* variant pathogenicity can be accomplished using trusted variant databases, published literature, functional studies, and predictive algorithms (Richards et al., 2015). While each assessment method can be useful, each has unique challenges that may lead to poor pathogenicity assessment. Databases may lack validation data, contain outdated information, or be based on small sample sizes. Published literature may reflect a poor study design or a limited sample size. Functional studies aim to understand the downstream effects of genetic variants (Rodenburg, 2018). For example, gene rescue assays seek to determine whether introducing the wild-type allele into patient derived cells “rescues” the phenotype. However, functional studies may not reflect the true biological environment or may not take full pathways into consideration (Richards et al., 2015). Common variant prediction algorithms, such as *SIFT* and *PolyPhen-2*, use nucleotide sequence homology (among other parameters) to predict whether protein function is affected by an amino acid substitution (Ng and Henikoff, 2003; Adzhubei et al., 2010). Predictive algorithms often have low specificity for missense variant prediction (Richards et al., 2015). Therefore, in most cases, it is important to use multiple programs for pathogenicity assessment (Niroula and Vihinen, 2019).

In the following sections, we provide an overview of studies that have identified *CH* variants in pediatric-cancer patients, provide insights into future directions in the field, and give a summary of available pediatric cancer sequencing data.

## Methods

### Literature Search

**Figure 2:**
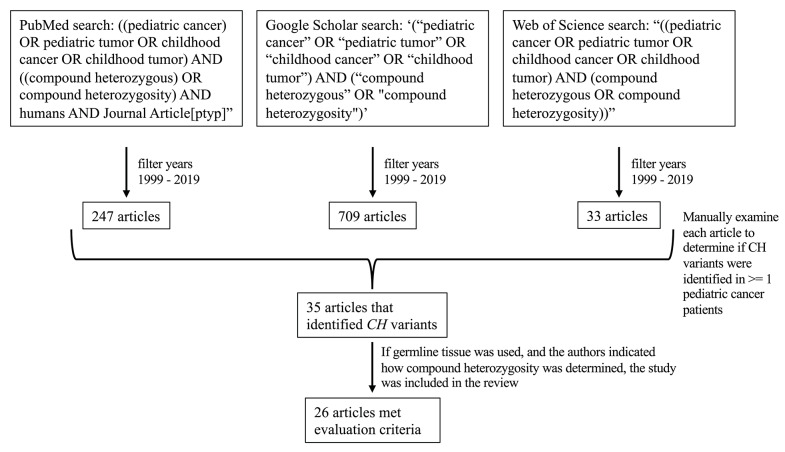
Flow diagram of how the studies in this review were identified. Twenty-six articles met the evaluation criteria.

On March 20, 2020, we searched PubMed, Google Scholar, and Web of Science for “((pediatric cancer) OR pediatric tumor OR childhood cancer OR childhood tumor) AND ((compound heterozygous) OR compound heterozygosity) AND humans AND Journal Article[ptyp],”, ‘(“pediatric cancer” OR “pediatric tumor” OR “childhood cancer” OR “childhood tumor”) AND (“compound heterozygous” OR “compound heterozygosity”),’ and “((pediatric cancer OR pediatric tumor OR childhood cancer OR childhood tumor) AND (compound heterozygous OR compound heterozygosity)),” respectively. The above searches were also filtered to be inclusive of studies published between 1999 and 2019. Based on these criteria, 247, 709, and 33 results were obtained from PubMed, Google Scholar, and Web of Science, respectively. Of the returned results, we examined each article's abstract (and full article text when necessary) to determine whether the researchers had identified *CH* variants in one or more pediatric cancer patients; we identified 35 total articles that met these criteria (Figure 2).

### Evaluation Criteria

We further evaluated the 35 articles to identify whether the authors had used germline tissue for DNA sequencing and described methods for assessing compound heterozygosity. If germline tissue was used for sequencing, and if the authors indicated how compound heterozygosity was determined, we included the study. Of the 35 studies identified, 26 met these criteria and are described in this review (Figure 2). For each of the 26 studies, we obtained additional information such as tumor type, the number of *CH* variants identified and the genes in which they were identified, the number of patients per cancer type that were included in the study, how compound heterozygosity was determined (i.e., whether through Mendelian inheritance or phasing), the type of sequencing technology used, the use of parental sequence data, and how variant pathogenicity was evaluated (Supplementary Data 1: T1).

### Tumor Type Standardization

To ensure consistency of names used to describe the tumor types and to reduce the total number of tumor types to consider, we standardized tumor type names, when possible, using parent terms defined in the National Cancer Institute Thesaurus (Sioutos et al., 2007). For example, we grouped B-cell acute lymphoblastic leukemia and T-cell acute lymphoblastic leukemia under their parent term, “acute lymphoblastic leukemia” (ALL).

### Variant Pathogenicity Reassessment and Cancer Pathway Association

Because pathogenicity estimation methods varied widely across the studies and because it had been many years since some articles were published, we reassessed pathogenicity using ClinVar and Variant Effect Predictor (VEP) for all studies that provided variant positions (McLaren et al., 2016; Landrum et al., 2018). We used Kyoto Encyclopedia of Genes and Genomes (KEGG) to determine which of the genes, across all studies, are in a known cancer pathway. We used Catalogue of Somatic Mutations in Cancer (COSMIC) to identify genes with known cancer associations (Kanehisa and Goto, 2000; Tate et al., 2019).

## Results

### Overview of Pediatric Cancer Types and Genes Studied

**Figure 3:**
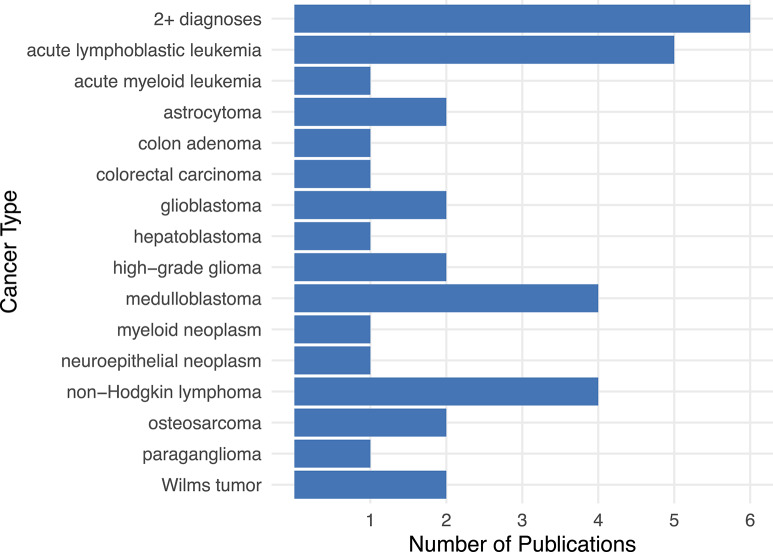
The number of publications per cancer type pertaining to *CH* variants in pediatric cancer. The literature on *CH* variants has covered a wide range of cancer types, especially acute lymphoblastic leukemia, non-Hodgkin's lymphoma, and medulloblastoma. In six publications, at least one patient was diagnosed with more than one cancer type. These “2+ diagnoses” include patients with the following cancer types: glioblastoma + non-Hodgkin's Lymphoma + oligodendroglioma (Bakry et al., 2014), glioblastoma + rectal carcinoma (Bakry et al., 2014), glioblastoma + non-Hodgkin's lymphoma (Chmara et al., 2013) or ALL + rectal adenoma (Herkert et al., 2011), acute myeloid leukemia + medulloblastoma (Scott et al., 2007), colon carcinoma + oligodendroglioma (De Rosa et al., 2000), brain tumor + rhabdomyosarcoma (Quesnel et al., 1999).

Researchers have identified *CH* variants across many pediatric cancer types, even though relatively few articles on these topics have been published overall. From 1999 until 2019, an average of ~1.2 journal articles per year were published on *CH* variant discovery across a total of 21 cancer types (Supplementary Data 1: T1); the highest number (*n* = 7) were published in 2018. The cancer types studied most frequently were ALL (5 publications) (Valentine et al., 2014; Spinella et al., 2015; Moriyama et al., 2017; Diets et al., 2018; Sharapova et al., 2018), non-Hodgkin's Lymphoma (4 publications) (Østergaard et al., 2005; Peters et al., 2009; Bakry et al., 2014; Diets et al., 2018), and medulloblastoma (4 publications) (Figure 3; Svojgr et al., 2016; Gröbner et al., 2018; Waszak et al., 2018; Schieffer et al., 2019). ALL and non-Hodgkin's lymphoma are both blood-based cancers, while medulloblastoma is a type of brain tumor (Sandlund et al., 1996; Hunger and Mullighan, 2015; Kumar et al., 2015). In addition, six publications described a patient having been diagnosed with two or more cancer types, which include: glioblastoma + non-Hodgkin's Lymphoma + oligodendroglioma (Bakry et al., 2014), glioblastoma + rectal carcinoma (Bakry et al., 2014), glioblastoma + non-Hodgkin's lymphoma (Chmara et al., 2013), ALL + rectal adenoma (Herkert et al., 2011), acute myeloid leukemia (AML) + medulloblastoma (Scott et al., 2007), colon carcinoma + oligodendroglioma (De Rosa et al., 2000), and brain tumor + rhabdomyosarcoma (Figure 3; Quesnel et al., 1999).

Sample sizes were limited in most studies (Table 1). Only ~19% of the studies had a sample size >10 for one or more cancer types (Valentine et al., 2014; Zhang et al., 2015; Diets et al., 2018; Gröbner et al., 2018; Waszak et al., 2018). Across all studies, cancer types with more than 10 samples included AML, ALL, high-grade glioma, medulloblastoma (Valentine et al., 2014; Zhang et al., 2015; Diets et al., 2018; Gröbner et al., 2018). Thirteen studies evaluated a single patient (Quesnel et al., 1999; De Rosa et al., 2000; Okkels et al., 2006; Scott et al., 2007; Peters et al., 2009; Majumdar et al., 2010; Chmara et al., 2013; Piane et al., 2016; Diness et al., 2018; Salih et al., 2018; Sharapova et al., 2018; Maciaszek et al., 2019; Schieffer et al., 2019).

**Table 1:** Genes with identified *CH* variants and the study(-ies) that identified them.

**Gene**	**Study(-ies)**
*ANKRD36*	Valentine et al. (AML: 13/13)
*ATM*	Sharapova et al. (ALL: 1/1); Piane et al. (astrocytoma: 1/1); Zhang et al. (HGG: 1/99)
*BRCA2*	Waszak et al. (MB: 4/1022); Gröbner et al. (MB: 1/42); Svojgr et al. (WT: 1/1, MB: 1/1)
*CEP55*	Spinella et al. (ALL: 2/2)
*DDX41*	Diness et al. (myeloid neoplasm: 1/1)
*DNAH2*	Spinella et al. (ALL: 2/2)
*ENOSF1*	Zhang et al. (HB: 2/2)
*FAM83H*	Diets et al. (ALL: 1/13)
*FLG*	Valentine et al. (AML: 3/13)
*GRIK1*	Diets et al. (ALL: 1/13)
*H1-2*	Diets et al. (NHL: 1/3)
*IRF5*	Diets et al. (WT: 1/1)
*KMT2C*	Valentine et al. (ALL: 6/12; AML: 13/13)
*MSH6*	Gröbner et al. (HGG: 2/67); Bakry et al. (NHL: 1/5; GB: 1/8); Peters et al. (NHL: 1/1); Scott et al. (MB + AML: 1/1); Okkels et al. (colorectal: 1/1); Østergaard et al. (NHL: 1/1; GB: 1/1);
*MUC4*	Zhang et al. (HB: 2/2)
*MYO18B*	Schieffer et al. (MB: 1/1)
*NUDT15*	Moriyama et al. (ALL: 1/5)
*PDE4DIP*	Spinella et al. (ALL: 2/2)
*PMS2*	Gröbner et al. (HGG: 1/67); Bakry et al. (NHL: 1/5; NHL + GB + oligodendroglioma: 1/1; rectal cancer + GB: 1/1); Chmara et al. (GB + NHL: 1/1); Herkert et al. (colon adenoma: 1/1; ALL + rectal adenoma: 1/1); Leenen et al. (NN: 1/1; astrocytoma: 1/1); De Rosa et al. (oligodendroglioma + colon carcinoma: 1/1)
*RBMX*	Valentine et al. (ALL: 6/12)
*RECQL4*	Maciaszek et al. (OS: 1/1); Salih et al. (OS: 1/1)
*RYR1*	Valentine et al. (AML: 9/13)
*SDHB*	Majumdar et al. (paraganglioma: 1/1)
*SLX4*	Spinella et al. (ALL: 2/2)
*TP53*	Quesnel et al. (rhabdomyosarcoma + brain tumor: 1/1)

*The details in parentheses next to the author name indicate the cancer type(s) associated with each CH variant, the number of patients evaluated in the study, and the number of patients who had a CH variant in that gene. For example, Valentine et al. evaluated 13 AML patients and identified a CH variant in all 13 patients for the ANKRD36 gene. AML, acute myeloid leukemia; ALL, acute lymphoblastic leukemia; HGG, high-grade glioma; MB, medulloblastoma; WT, Wilms tumor; HB, hepatoblastoma; NHL, non-Hodgkin's lymphoma; GB, glioblastoma; NN, neuroepithelial neoplasm; OS, osteosarcoma*.

Across all studies, at least one *CH* variant was identified in 25 genes (Table 1). Of these genes, 7 are known to be associated with hereditary cancer predisposition (Table 2). The remaining 18 genes may provide clues about alternative mechanisms of cancer predisposition. For example, one study identified 6 ALL and 13 AML patients with a *CH* variant in *KMT2C* (Valentine et al., 2014), and one study identified two hepatoblastoma patients with a *CH* variant in *MUC4* (Zhang et al., 2018). DNA alterations in *KMT2C* and *MUC4* have been observed in the somatic tissue of medulloblastoma and head and neck squamous cell carcinoma patients, respectively (Tate et al., 2019). Therefore, future studies may reveal that germline mutations in these genes also contribute to pediatric cancer predisposition.

**Table 2:** Details of genes with identified *CH* variants.

**Gene**	**Gene CDS length**	**Total gene length**	**Cancer pathway (KEGG)**	**Germline tumor types (COSMIC)**	**Role in cancer (COSMIC)**
*ANKRD36*	5,678	151,216	–	–	–
*ATM*	9,272	146,618	–	Leukemia; lymphoma; medulloblastoma; glioma	TSG
*BRCA2*	10,415	85,180	Yes	Breast; ovarian; pancreatic; leukemia	TSG
*CEP55*	1,454	32,480	–	–	–
*DDX41*	1,802	5,384	–	–	–
*DNAH2*	13,320	116,390	–	–	–
*ENOSF1*	1,337	42,344	–	–	–
*FAM83H*	5,192	14,301	–	–	–
*FLG*	12,181	23,074	–	–	–
*GRIK1*	2,887	403,100	–	–	–
*H1-2*	638	641	–	–	–
*IRF5*	1,493	12,581	–	–	–
*KMT2C*	15,426	301,083	–	–	TSG
*MSH6*	3,591	114,571	Yes	Colorectal; endometrial; ovarian	TSG
*MUC4*	10,911	65,208	–	–	Oncogene
*MYO18B*	7,889	288,897	–	–	–
*NUDT15*	489	9,495	–	–	–
*PDE4DIP*	7,104	240,105	–	–	Fusion
*PMS2*	2,463	38,181	–	Colorectal; endometrial; ovarian; medulloblastoma; glioma	TSG
*RBMX*	1,166	32,760	–	–	–
*RECQL4*	3,252	6,557	–	Osteosarcoma; skin basal cell; skin squamous cell	Oncogene; TSG
*RYR1*	14,835	153,873	–	–	–
*SDHB*	778	35,310	–	Paraganglioma; pheochromocytoma	TSG
*SLX4*	5,488	30,425	–	–	–
*TP53*	1,194	25,771	Yes	Breast; sarcoma; adrenocortical carcinoma; glioma; multiple other tumor types	Oncogene; TSG; fusion

*The average CDS gene length across all studies was 5,610 and the average total gene length was 95,022. ATM, BRCA2, MSH6, PMS2, RECQL4, SDHB, and TP53 are all associated with germline tumor types and are known tumor suppressor genes as classified by COSMIC. BRCA2, MSH6, and TP53 are all part of a cancer pathway as classified by KEGG. A value of “–” indicates that an association was not observed. TSG, tumor suppressor gene*.

### Overview of Studies that Identified CH Variants in Genes Associated With Pediatric Cancers

Here we provide an overview of *CH* variant findings specific to genes that have a known association to cancer predisposition. Of the 25 characterized genes identified across all studies (Table 1), COSMIC classifies 7 as being associated with hereditary predisposition for at least one type of pediatric cancer: *ATM, BRCA2, MSH6, PMS2, RECQL4, SDHB*, and *TP53* (Table 2). Below we provide insight about the functions of these genes, prior associations that have been made for non-compound germline variants, and *CH* variants in these genes.

*ATM, BRCA2*, and *SDHB* are known tumor suppressor genes. Non-compound germline variations in these genes have been associated with leukemias, lymphomas, medulloblastomas, and gliomas (Table 2). Similarly, *CH* variants in *ATM* have been observed in ALL, astrocytoma, and high-grade glioma (Zhang et al., 2015; Piane et al., 2016; Sharapova et al., 2018). *BRCA2* plays important roles in DNA repair, and germline variations in this gene have been associated with breast cancer, ovarian cancer, pancreatic cancer, and leukemia risk (Table 2; Kanehisa and Goto, 2000; Tate et al., 2019). To date, *CH* variants in *BRCA2* have been observed in medulloblastomas and Wilms tumors (Svojgr et al., 2016; Gröbner et al., 2018; Waszak et al., 2018). Variations in *SDHB* have been associated with paraganglioma and pheochromocytoma (Table 2); one patient with paraganglioma had a *CH* variant in this gene (Majumdar et al., 2010).

*MSH6* and *PMS2* are both considered to be tumor-suppressor and mismatch repair (MMR) genes (Ripperger and Schlegelberger, 2016; Tabori et al., 2017; Tate et al., 2019). Patients with biallelic germline mutations in an MMR gene (*MLH1, MSH2, MSH6*, and *PMS2*) are considered to have a syndrome known as constitutional mismatch repair disease (CMMRD) (Ripperger and Schlegelberger, 2016; Tabori et al., 2017). Germline mutations in MMR genes have been associated with a predisposition to many different types of cancer (Table 2; Tabori et al., 2017), including hematological malignancies, brain tumors, and digestive tract cancers (Ripperger and Schlegelberger, 2016). To date, *CH* variants in *MSH6* have been identified in patients with high-grade glioma, non-Hodgkin's lymphoma, glioblastoma, medulloblastoma, AML, or colorectal cancer (Østergaard et al., 2005; Okkels et al., 2006; Scott et al., 2007; Peters et al., 2009; Bakry et al., 2014; Gröbner et al., 2018). *PMS2 CH* variants have been identified in high-grade glioma, non-Hodgkin's lymphoma, glioblastoma, oligodendroglioma, rectal cancer, colon adenoma, ALL, recta adenoma, neuroepithelial neoplasm, astrocytoma, and colon carcinoma (De Rosa et al., 2000; Herkert et al., 2011; Leenen et al., 2011; Chmara et al., 2013; Bakry et al., 2014; Gröbner et al., 2018).

*RECQL4* and *TP53* both have more than one role in cancer (Tate et al., 2019). *RECQL4* is involved in many intracellular regulatory pathways and can act either as an oncogene or a tumor suppressor gene (Kellermayer, 2006; Arora et al., 2016). Germline cancer associations for *RECQL4* include osteosarcoma, skin basal cell, and skin squamous cell (Table 2). *CH* variants in *RECQL4* have been associated with osteosarcoma in two studies (Salih et al., 2018; Maciaszek et al., 2019). *TP53* is involved in many cancer pathways, and germline variation in this gene has been associated with a wide range of tumor types (Table 2). One patient with rhabdomyosarcoma + brain tumor had a *CH* variant in *TP53* (Quesnel et al., 1999).

### Methodologies Used to Identify CH Variants and Assess Pathogenicity

The sequencing methods used were highly variable across the studies (Table 3 and Supplementary Data 1:T1). Of the studies in this review, 10 indicated that they used one or more forms of NGS sequencing (whole-exome, whole-genome, RNA-seq) and thus were able to sample *CH* variants broadly (Valentine et al., 2014; Spinella et al., 2015; Zhang et al., 2015, 2018; Diets et al., 2018; Diness et al., 2018; Gröbner et al., 2018; Waszak et al., 2018; Maciaszek et al., 2019; Schieffer et al., 2019). Thirteen studies indicated that they used non-NGS methods (Sanger, Direct sequencing, SNP-array, Multiplex ligation-dependent probe amplification) and were therefore limited in the number of genes and *CH* variants analyzed (Quesnel et al., 1999; De Rosa et al., 2000; Østergaard et al., 2005; Okkels et al., 2006; Scott et al., 2007; Herkert et al., 2011; Leenen et al., 2011; Chmara et al., 2013; Bakry et al., 2014; Piane et al., 2016; Svojgr et al., 2016; Moriyama et al., 2017; Sharapova et al., 2018). Three clinical/case report studies did not describe the exact DNA sequencing technology used (Peters et al., 2009; Majumdar et al., 2010; Salih et al., 2018); however, we inferred that Sanger sequencing was inferred in these three studies because single genes were the focus of the studies, parent DNA was also sequenced, and DNA variant locations were provided by the authors.

**Table 3:** Methods used by each study for identification and evaluation of *CH* variants.

**References**	**Sequencing technology**	**How *CH* variant identified**	**Post sequencing pathogenicity evaluation criteria**
Maciaszek et al. (2019)	WGS	Mendelian inheritance	ACMG/AMP guidelines
Schieffer et al. (2019)	WES; Sanger	Mendelian inheritance	ACMG/AMP guidelines
Zhang et al. (2018)	WGS	Mendelian inheritance	*SIFT; PolyPhen-2; MutationTaster* (Schwarz et al., 2014), M-CAP; and AMP/ACMG guidelines
Diness et al. (2018)	WES	Mendelian inheritance	*CADD* (Kircher et al., 2014); gene expression analysis
Sharapova et al. (2018)	Sanger	Mendelian inheritance	*PolyPhen-2*
Waszak et al. (2018)	WGS; WES; RNA-seq	Multiple sites within the same gene were phased with paired-end RNA sequencing data and individual sites were merged to calculate haplotype-specific expression ratios.	ClinVar
Diets et al. (2018)	WES	Mendelian inheritance	*SIFT; PolyPhen2; CADD*
Gröbner et al. (2018)	WGS; WES	Used Platypus which is a haplotype-based variant caller (Rimmer et al., 2014)	*CADD; Mutation Assessor* (Reva et al., 2011)
Salih et al. (2018)	Sanger (inferred)	Mendelian inheritance	–
Moriyama et al. (2017)	Sanger	PHASE was used to infer haplotypes (Stephens and Scheet, 2005)	–
Svojgr et al. (2016)	SNP-array	Mendelian inheritance	–
Piane et al. (2016)	Sanger	Mendelian inheritance	*SIFT; Polyphen; MutationTaster*
Spinella et al. (2015)	WES	Mendelian inheritance	*SIFT; PolyPhen-2;* and hidden Markov models
Zhang et al. (2015)	WGS; WES; RNA-seq	Used RNA-seq data to determine *CH* nature of variant	ACMG/AMP guidelines; genetic database; medical literature; computational predictions; and second hits identified in the tumor genome
Valentine et al. (2014)	WES	Mendelian inheritance	Filtered for functional consequences (e.g., non-synonymous and coding)
Bakry et al. (2014)	Sanger	Mendelian inheritance	Algorithms to predict RNA/protein disruption
Chmara et al. (2013)	Direct sequencing; MLPA	Mendelian inheritance	–
Herkert et al. (2011)	Direct sequencing; MLPA	Mendelian inheritance	*SIFT; AlignGVGD* (Tavtigian et al., 2008); *PolyPhen-2*; and RNA splice site prediction programs
Leenen et al. (2011)	Sanger	Mendelian inheritance	Literature search
Majumdar et al. (2010)	Sanger (inferred)	Mendelian inheritance	–
Peters et al. (2009)	Sanger (inferred)	Mendelian inheritance	–
Scott et al. (2007)	Sanger	Mendelian inheritance	Literature search
Okkels et al. (2006)	Sanger	Mendelian inheritance	–
Østergaard et al. (2005)	Sanger	Mendelian inheritance	–
De Rosa et al. (2000)	Sanger	Mendelian inheritance	–
Quesnel et al. (1999)	Sanger	Mendelian inheritance	mRNA assay

*A value of “–” indicates that no clear description was provided by the authors. WGS, whole genome sequencing; WES, whole exome sequencing; MLPA, Multiplex Ligation-dependent Probe Amplification*.

Across all studies, the methodology used to estimate haplotypes varied considerably (Table 3 and Supplementary Data 1:T1). Four of the studies used a computer-based phasing algorithm to estimate haplotypes (Zhang et al., 2015; Moriyama et al., 2017; Gröbner et al., 2018; Waszak et al., 2018). One study used evidence from RNA-seq data that the alternate alleles were on different chromosomes (Zhang et al., 2015). All other studies that performed phasing used sequence data from the patient and his/her parent(s) (i.e., Mendelian inheritance; Quesnel et al., 1999; De Rosa et al., 2000; Okkels et al., 2006; Scott et al., 2007; Peters et al., 2009; Majumdar et al., 2010; Herkert et al., 2011; Leenen et al., 2011; Chmara et al., 2013; Bakry et al., 2014; Valentine et al., 2014; Spinella et al., 2015; Piane et al., 2016; Svojgr et al., 2016; Diets et al., 2018; Diness et al., 2018; Salih et al., 2018; Sharapova et al., 2018; Zhang et al., 2018; Maciaszek et al., 2019; Schieffer et al., 2019).

The methods used to classify variant pathogenicity also varied widely from study to study (Table 3 and Supplementary Data 1:T1). Eight studies used predictive algorithms as the sole means of pathogenicity assessment (Herkert et al., 2011; Bakry et al., 2014; Spinella et al., 2015; Piane et al., 2016; Diets et al., 2018; Gröbner et al., 2018; Sharapova et al., 2018; Waszak et al., 2018). Four studies used guidelines set forth by the American College of Medical Genetics and Genomics (ACMG) and the Association for Molecular Pathology (AMP) (Zhang et al., 2015, 2018; Maciaszek et al., 2019; Schieffer et al., 2019). Two studies used literature searches to look for known effects of the identified variants (Scott et al., 2007; Leenen et al., 2011). Two studies analyzed RNA expression of altered alleles (Quesnel et al., 1999; Diness et al., 2018). One study did not directly assess pathogenicity; rather they reported on variants that met specific criteria such as the variant being a non-synonymous substitution (Valentine et al., 2014). Nine studies made no mention of assessing *CH* variant pathogenicity (De Rosa et al., 2000; Østergaard et al., 2005; Okkels et al., 2006; Peters et al., 2009; Majumdar et al., 2010; Chmara et al., 2013; Svojgr et al., 2016; Moriyama et al., 2017; Salih et al., 2018).

There were a total of 18 *CH* variants where both alleles were reported as pathogenic or likely pathogenic by the authors of the study (Supplementary Data 1:T2). Because some of the studies were conducted years ago and pathogenicity classifications may have been updated, we re-assessed the pathogenicity of all variants that were provided by the authors of the studies. Using ClinVar and VEP (as SIFT and PolyPhen scores), we were able to confirm pathogenicity for 5 of the 18 *CH* variants with one or more of our reassessment methods (Supplementary Data 1:T2). These confirmed variants were in *MYO18B* (Schieffer et al., 2019), *BRCA2* (Waszak et al., 2018), *FAM83H* (Diets et al., 2018), and *HIST1H1C* (Diets et al., 2018). In addition, we were able to classify 3 *CH* variants as pathogenic or likely pathogenic that were not indicated as such in the original study. These variants were in *RECQL4* (Salih et al., 2018), *PMS2* (Herkert et al., 2011), and *MSH6* (Scott et al., 2007). For the remaining variants, either only one allele from a *CH* pair was able to be classified, or no information was available in the databases (Supplementary Data 1:T2).

## Discussion

### Observations and Future Directions

Research to date has highlighted that *CH* variants are observed relatively rarely but occur in many different genes and a diverse array of pediatric tumor types. However, our knowledge of the roles that *CH* variants play in pediatric cancers is only in its infancy. Prior studies have focused primarily on candidate genes, have been limited to individual cancer types, or have been limited by small sample sizes (Table 1). Due to these issues, important *CH* variants may have been missed. For example, in AML, tumor suppressor genes that are known to harbor germline risk variants include *BRIP1, FANCA, FANCC, FANCD2, FANCE, FANCF, FANCG, PALB2*, and *SBDS* (Sondka et al., 2018; Tate et al., 2019). However, no study to date has found *CH* variants in any of these genes for AML patients. Similarly, *ATM, NBN, PALB2, PMS2, PTCH1*, and *SUFU* are tumor suppressor and germline risk genes for medulloblastoma, but *CH* variants in these genes have not been found in prior studies.

One factor that may have contributed to a lack of identifying *CH* variants in known cancer-predisposition genes may be filtering based on minor allele frequencies (MAF). Commonly, individual alleles with a MAF >1% (or >0.5%) in a control population are excluded from analyses because they are assumed to have benign effects (Richards et al., 2015; Niroula and Vihinen, 2019). Yet, this traditional filtering criterion may be too stringent for identifying pathogenic *CH* variants. By definition, two pathogenic alleles must be present for a recessive phenotype to manifest itself. Suppose, for example, that one allele in a given gene is relatively rare, having been observed in 0.8% of the population. Now suppose that a second allele is present at a different locus within the same gene and that this allele has a population prevalence of 5%. MAF filtering considers each locus separately, so the latter variant would be excluded by the 1% threshold, causing this potentially pathogenic *CH* variant to be overlooked. Assuming non-consanguinity and using the probability multiplication rule, the population prevalence of this particular *CH* variant would be estimated at approximately 0.04% (5% x 0.8%). Although care must be taken to consider other possible combinations of *in trans* alleles in this gene (Eilbeck et al., 2017), this example illustrates that traditional MAF filtering may be too simplistic for *CH* variant analysis. Furthermore, researchers must account for any *homozygous*, non-reference genotypes that have been observed for either allele in healthy individuals (Kamphans et al., 2013).

Further complicating matters, it is difficult to estimate the *a priori* expectation of finding a *CH* variant in a given gene. The longer a gene, the higher the probability that two pathogenic alleles will occur within that gene. Accordingly, genome-wide studies may be biased toward identifying *CH* variants in longer genes. For example, across the 26 studies covered in this review, the average coding sequence (CDS) length for genes with an identified *CH* variant was 5,610 bases (median: 3,591 bases) (Table 2). Comparatively, using data from GENCODE, we calculated the average CDS length of protein coding genes across the human genome to be ~1,796 bases (median: 1,338) (Frankish et al., 2019). Efforts should be made to help alleviate this bias and other confounding factors. For example, Itan et al. developed the Gene Damage Index to prioritize genes based on CDS length, protein complexity, paralog count, and evolutionary pressures (Itan et al., 2015). When prioritizing *CH* variants at the gene level in this way, the number of false-positive genes may be reduced.

### Available Pediatric Cancer Data

The future is primed for more rapid discovery of genetic factors involved in pediatric cancers and a clearer understanding of the roles that *CH* variants play in pediatric cancer development and progression. Publicly available data are becoming readily accessible for researchers to study, and more data will become available over the next few years. For example, the NIH-funded Gabriella Miller Kids First (GMKF) initiative is sequencing germline DNA from hundreds of trios (affected child and both parents). This initiative is enabling researchers to obtain Illumina-based, whole-genome sequencing (WGS) data for these trios across many childhood cancer types (and other pediatric diseases) (Gabriella Miller Kids First Pediatric Research Program^2^). Currently, the GMKF repository contains sequencing data for Ewing sarcoma (250 trios) and neuroblastoma (470 trios). In the coming years, the GMKF repository is expected to include data from pediatric cancers such as osteosarcoma, leukemia, enchondromatosis, brain tumors, myeloid malignancies, and others. This repository will prove valuable as parental data may allow for better identification of *CH* variants and *de novo* mutations (Francioli et al., 2017; Choi et al., 2018).

Frequently it is infeasible to obtain sequencing data from a proband's parents due to cost limitations or logistical challenges (a parent may not consent to participate or may be unavailable, a parent may be deceased, the child may be adopted, etc.). Projects such as Therapeutically Applicable Research to Generate Effective Treatments (TARGET), St. Jude Cloud, and the Children's Brain Tumor Tissue Consortium (CBTTC) have sequenced DNA for thousands of patients across many pediatric diseases, but sequencing data are available for the proband only in these resources (Children's Hospital of Philadelphia^3^; Downing et al., 2012; Office of Cancer Genomics, 2013). At the time of this writing, TARGET included matched tumor-normal, multi-omic data across six types of pediatric cancer, including AML (*n* ≈ 50), neuroblastoma (*n* ≈ 228), Wilms tumor (*n* ≈ 81), osteosarcoma (*n* ≈ 89), clear cell sarcoma of the kidney (*n* ≈ 13), and rhabdoid tumor (*n* ≈ 43) (Office of Cancer Genomics, 2013). The St. Jude Cloud contained matched tumor-normal data for ~42 pediatric cancer types/subtypes, which encompassed ~2,168 patients (Downing et al., 2012). Some of the datasets with the highest number of patients in the St. Jude Cloud included ALL (*n* ≈ 260), AML (*n* ≈ 189), HGG (*n* ≈ 163), and neuroblastoma (*n* ≈ 135) (Downing et al., 2012). Eight cancer types are represented by over 100 patients, while 14 cancer types had data for 10 or fewer patients. The CBTCC is a collaborative effort dedicated to the study and treatment of pediatric brain tumors (Children's Hospital of Philadelphia). At the time of this writing, CBTCC included genomic data for ~871 patients across ~38 pediatric brain tumor types. Although these databases do not contain parental genome data, computer-based algorithms can often determine a variant's parent-of-origin using haplotype reference panels (Browning and Browning, 2011).

Thanks to these public and non-profit efforts, we have entered an era in which researchers can shed more light on the genes and pathways that influence specific pediatric cancers through the use of genome-wide, publicly available data. We advocate that researchers take advantage of these resources.

## Conclusion

Many discoveries about *CH* variants and their association with pediatric cancers have been made over the last 20 years; the role of *CH* variants in cancer and developmental pathways and the prevalence of these variants in pediatric cancers are beginning to be uncovered. Through the works discussed in this review, much insight has been gained. As future studies are conducted on *CH* variants in pediatric cancers, we anticipate that *CH* variants will play a more prominent role in elucidating disease mechanisms. This heightened knowledge could expand this field of study and eventually lead to the development of targeted treatments. Furthermore, having an understanding of *CH* variants and their role in disease development could prove beneficial for disease monitoring. For example, a child with a malignancy who has a germline risk variant in a known predisposition gene and/or key pathway could be more regularly monitored and assessed for additional tumor development (Milanese and Wang, 2019). If risk variants are in genes associated with one particular type of cancer, screening efforts could be more directed than general cancer screening. More specific screening could allow for earlier detection of tumors, sooner treatments, and prophylactic measures. Finally, a heightened knowledge of *CH* variants could lead to an expansion of our understanding of other pediatric diseases, such as birth defects, and even inherited disorders that arise in adults.

## Author Contributions

DM wrote the manuscript and performed critical analysis. SP provided edits and critical feedback.

## Conflict of Interest

The authors declare that the research was conducted in the absence of any commercial or financial relationships that could be construed as a potential conflict of interest.

## Footnotes

^1^*American Cancer Society Risk Factors and Causes of Childhood Cancer*. Available online at: https://www.cancer.org/cancer/cancer-in-children/risk-factors-and-causes.html (accessed March 29, 2019).

^2^*Gabriella Miller Kids First Pediatric Research Program National Institutes of Health Office of Strategic Coordination*- *The Common Fund*. Available online at: https://commonfund.nih.gov/kidsfirst (accessed March 26, 2019).

^3^Children's Hospital of Philadelphia Children's Brain Tumor Tissue Consortium. *Children's Brain Tumor Tissue Consortium*. Available online at: https://cbttc.org/ (accessed January 31, 2020).
